# Supplementary material for: Global translational control by the transcriptional repressor TrcR in the filamentous cyanobacterium Anabaena sp. PCC 7120
Source: Commun Biol. 2023 Jun 15;6:643. doi: 10.1038/s42003-023-05012-9 (PMC10272220; doi:10.1038/s42003-023-05012-9)
Supplement: Supplementary file 2 — Supplementary Information [file 42003_2023_5012_MOESM2_ESM.pdf]

Supplementary Information

Supplementary Table 1. Mutations mapped in different BMAA^r mutants. BMAA^r: BMAA-resistance; -: no mutation detected in the corresponding ORF.

**BMAA^r strains**	**Mutated ORF**			
	alr4167	***all1284***	***all0854***	***alr2300***
M1	deletion of A354	A716G (Tyr239Cys)	-	-
M2	C253T (R85 stop codon)	C80T (Thr27Ile)	-	-
M3	-	deletion of A652	-	-
M4	-	-	-	-
M5	-	A716G (Tyr239Cys)	-	-
M6	-	-	-	-
M7	-	-	-	-
M8	T8C (Met3Thr)	-	-	-
M9	C253T (R85 stop codon)	insertion of TGG203	-	-
M10	C253T (R85 stop codon)	-	-	-
M11	G263A (Gly88Glu)	deletion of A652	-	-
M12	-	deletion of A652	-	-
M13	insertion of GG127	-	-	-
M14	-	-	-	-
M15	C217T (Leu73Phe)	deletion of A652	-	-
M16	-	deletion of A649	-	-
M17	-	-	-	G137A (Gly46Asp)
M18	-	-	C215A (Ala72Asp)	-
M19	-	-	T131C (Leu44Pro)	-
M20	-	-	T131C (Leu44Pro)	-

Supplementary Table 2. Cyanobacterial strains used in this study. Nm^{r}: neomycin resistance.

**Stains**	**Description**	**Source**
Anabaena PCC 7120	Wild type (WT)	Pasteur Culture collection
Δ*trcR*	A markerless mutant with deletion of an internal fragment from position 10 to 333 within the ORF of *all0854*/*trcR*	This study
Δ*trcR*Δ*alr1537*	A markerless double mutant of *all0854* and *alr1537*	This study
Δ*trcR*Δ*alr1538*	A markerless double mutant of *all0854* and *alr1538*	This study
Δ*trcR*Δ*alr1539*	A markerless double mutant of *all0854* and *alr1539*	This study
Δ*trcR*Δ*alr1540*	A markerless double mutant of *all0854* and *alr1540*	This study
Δ*trcR*Δ*alr1537-40*	A markerless quintuple mutant of *all0854*, *alr1537*, *alr1538*, *alr1539* and *alr1540*	This study
C-*trcR*	Nm^{r}; Δ*trcR* strain complemented by pRL-Call0854	This study
M20-C*trcR*	Nm^{r}; M20 strain complemented by pRL-Call0854	This study
WT::p$_{trcR}$CFP	Nm^{r}; WT strain expressing plasmid-born *all0854* transcriptional fusion.	This study
M20::p$_{trcR}$CFP	Nm^{r}; M20 strain expressing plasmid-born *all0854* transcriptional fusion.	This study
Δ*trcR*::p$_{trcR}$CFP	Nm^{r}; Δ*trcR* strain expressing plasmid-born *all0854* transcriptional fusion.	This study
WT::p$_{CT}$TrcR-p$_{trcR}$CFP	Nm^{r}; WT strain expressing plasmid-born *all0854* transcriptional fusion and *all0854* (under the control of CT promoter) from the same plasmid[1].	This study
Δ*trcR*::p$_{CT}$TrcR-p$_{trcR}$CFP	Nm^{r}; Δ*trcR* strain expressing plasmid-born *all0854* transcriptional fusion and *all0854* (under the control of CT promoter) from the same plasmid[1].	This study
WT::p$_{trn}$CFP	Nm^{r}; WT strain expressing plasmid-born *trn* transcriptional fusion.	This study
WT::p$_{alr3301}$CFP	Nm^{r}; WT strain expressing plasmid-born *alr3301* transcriptional fusion.	This study
WT::p$_{all3526}$CFP	Nm^{r}; WT strain expressing plasmid-born *all3526* transcriptional fusion strain.	This study
WT::p$_{alr8077}$CFP	Nm^{r}; WT strain expressing plasmid-born *alr8077* transcriptional fusion.	This study

Supplementary Table 3. Primers for qRT-PCR

Gene	Primer	Sequence (5’ to 3’)
allrs04 (reference gene)	Pallrs04F334	CCAGTTCCGCTATCAGAGAG
	Pallrs04R456	GAGGAGAGAGTTGGTGGTAAG
all3526	Pall3526F231	TTGCGGTATGAGTGCGAT
	Pall3526R378	ACGTTGCCAGTTAGCCAC
alr3303	Palr3303F576	TCAGTTCCAGCAGTATATC
	Palr3303R683	GCTACATAACGGTAATCATC
alr8077	Palr8077F241	TGTGCTACCATTCACGGA
	Palr8077R368	CCATCAAGTCCACAAACC
alr1537	Palr1537F585	TCGCACACTTGATACAGAA
	Palr1537R677	TTGGTTCGCTCTTGAGTAA
alr1538	Palr1538F101	AAGCACTTGATGTGTATACAGT
	Palr1538R239	GCGATCGCTAATAACTTCCA
alr1539	Palr1539F80	GGATACGACAAAGGTTAGG
	Palr1539R189	TTGATGAGAATGCCAGTG
alr1540	Palr1540F190	ACTGCTGATGGAATTGTTG
	Palr1540R289	TTGGGTCGTAGTTGAGAG
int (an interval fragment in *trn*)	Int2-1F[2]	CATGGGGTGACGAATGGTGATC
	Int2-1R[2]	AAGGGGAATCGAACCCCTATC
Leu-tRNA	tRNALeu3-1F[2]	ACCCCTGTGATGCAATTGGC
	tRNALeu-TAA-1R[2]	GGATTTGAACCTGTAGAAAACCCG
Val-tRNA	tRNAVal-1F[2]	AGCCCTGTAACTCAATTGGT
	tRNAVal-2R[2]	CAGGATTTGAACCCGCTAC
Gln-tRNA	tRNAGln-TTG-1F[2]	GGAAGTAGGCGACGTTGGC
	tRNAGln-TTG-1R[2]	GGAATCGAACCCGCGAG
allrt06	Pallrt06F6	GTTAGCTCAGTTGGTAGA
	Pallrt06R68	AGTACCAGACTCGAACTG
allrt16	Pallrt16F13	AAGTGGTAAGGCATCGGGTTT
	Pallrt16R72	CTGGGGCGCTAGGATTCG
allrt02	Pallrt02F7	GTGGCGAAATTGGTAGAC
	Pallrt02R74	GAGAATCGAACTCCTATGAC
all0854	Pall0854F240	CACAGGTCAACTCATCCA
	Pall0854R315	ACTGAGCGGAACCATATC

Supplementary Table 4. Primers for plasmid construction

Plasmid	Primer	Sequence (5’ to 3’)	Description
pCpf1b-Mall0854R246	Pall0854F1151m	GCAGAAATTCGATATCTAGATCTGCTTCTGCTTTTAGCGCTTC	For upstream fragment amplification
	Pall0854R9	TGTGTGCATGATTTTTCTCC	
	Pall0854F334	AGAAAAATCATGCACACATAGTTTTACATTTGTCCGAATACAG	For downstream fragment amplification
	Pall0854R1553	AACGTTGTTGCCATTGCGGATCCTGAATCATCCCAATCATGAGA	
	cr_all0854R246F	AGATACCTGTGAGTACACAACGTTCT	Annealed for guide sequence
	cr_all0854R246R	AGACAGAACGTTGTGTACTCACAGGT	
pCpf1b-Malr1537R372	Palr1537F1310m	GCAGAAATTCGATATCTAGAATTCTTACATGATTGTGATAGAGAA	For upstream fragment amplification
	Palr1537R6	AAGCATGAGTTACAATCATACTAC	
	Palr1537F1178	GTAGTATGATTGTAACTCATGCTTTGGATGATTGATTTCTTTTAATTAGG	For downstream fragment amplification
	Palr1537R2441	AACGTTGTTGCCATTGCGGAAACTCATCTTGATGAGAATGC	
	Cr-alr1537R372F	AGATTGTAGCTGGATAGAGAACCGAG	Annealed for guide sequence
	Cr-alr1537R372R	AGACCTCGGTTCTCTATCCAGCTACA	
pCpf1b-Malr1538F404	Palr1538F1347m	GCAGAAATTCGATATCTAGAAATCGGGTGATTCTACAGAA	For upstream fragment amplification
	Palr1538R15	AGAACTGTGGTGCATAATAATTCG	
	Palr1538F910	CGAATTATTATGCACCACAGTTCTGCTTCATAGTCTAAAATCTACAAC	For downstream fragment amplification
	Palr1538R2248	AACGTTGTTGCCATTGCGGAGCAACAACTGTTCCGCAA	
	Cr-alr1538F404F	AGATGTATATTAACTTTAGGTTACGT	Annealed for guide sequence
	Cr-alr1538F404R	AGACACGTAACCTAAAGTTAATATAC	
pCpf1b-Malr1539R169	Palr1539F1193m	GCAGAAATTCGATATCTAGATTTATCTTATCGGTTGCACA	For upstream fragment amplification
	Palr1539R9	GATAATCATAAGATTATTAGCTAGTAAAC	

	Palr1539F451	CTAGCTAATAATCTTATGATTATCGGAACGCGATATAAATAGGGT	For downstream fragment amplification
	Palr1539R1735b	AACGTTGTTGCCATTGCGGAGTGTCGATGAAATCGTCTGTA	
	Cr-alr1539F169F	AGATAGACACTGGCATTCTCATCAAG	Annealed for guide sequence
	Cr-alr1539F169R	AGACCTTGATGAGAATGCCAGTGTCT	
pCpf1b-Malr1540R179	Palr1540F1208m	GCAGAAATTCGATATCTAGACAATTAGCTCCCCTCTTGTT	For upstream fragment amplification
	Palr1540R21	TTGAGCATTAGCGTTAGTCAT	
	Palr1540F382b	ATGACTAACGCTAATGCTCAACAGTAGGTTGAAAAGTAAGAGTT	For downstream fragment amplification
	Palr1540R1651	AACGTTGTTGCCATTGCGGATATAGTGACCACCAAGCAG	
	Cr-alr1540R179F	AGATGCGCGCTGAAATTTGATCACTG	Annealed for guide sequence
	Cr-alr1540R179R	AGACCAGTGATCAAATTTCAGCGCGC	
pCpf1b-Malr1537-40R674	Palr1537F1310m	GCAGAAATTCGATATCTAGAATTCTTACATGATTGTGATAGAGAA	For upstream fragment amplification
	Palr1537R6	AAGCATGAGTTACAATCATACTAC	
	Palr1540F382	GTATGATTGTAACTCATGCTTCAGTAGGTTGAAAAGTAAGAGTT	For downstream fragment amplification
	Palr1540R1651	AACGTTGTTGCCATTGCGGATATAGTGACCACCAAGCAG	
	Cr-alr1537R674F	AGATGTTCGCTCTTGAGTAAAGATGA	Annealed for guide sequence
	Cr-alr1537R674R	AGACTCATCTTTACTCAAGAGCGAAC	
pRL-Call0854	PpCT-R2979	TCCAAAAAAAAACCCCGCCGAA	For vector amplification
	PpCT-F3530	GGGGGTTCTGGTGGTGGTAG	
	Pall0854F100m	CGGCGGGGTTTTTTTTTGGATGGGGTAAATGGATTTCACA	For wide-type gene *all0854* amplification
	Pall0854R339m	CTACCACCACCAGAACCCCCAAACTAGCCGACTAAACTCTC	
Transcriptional fusion plasmid	PpCT-R2979	TCCAAAAAAAAACCCCGCCGAA	For vector amplification
	PV_16	GGTGGATCTGGAGGTAGTGGT	
p_{trcR}CFP	Pall0854F100m	CGGCGGGGTTTTTTTTTGGATGGGGTAAATGGATTTCACA	*all0854* promoter amplification

	Pall0854R18	ACCACTACCTCCAGATCCACCG CGAGCGATTGTGTGCAT	
$p_{alr1537}$CFP	Palr1537F515m	CGGCGGGGTTTTTTTTTGGAGT ATTGGAATTTGTCTCTGGTG	*alr1537* promoter amplification
	Palr1537R24	ACCACTACCTCCAGATCCACCC ATTGGTTCTACCATTAGAAGCAT	
$p_{alr3301}$CFP	Palr3301F515m	CGGCGGGGTTTTTTTTTGGAC GTAAAGATTTCTATGCCACCAT	*alr3301* promoter amplification
	Palr3301R18	ACCACTACCTCCAGATCCACCG TCAATTAGGTCTGTCATACA	
$p_{alr8077}$CFP	Palr8077F572m	CGGCGGGGTTTTTTTTTGGAC GCTGCTAGTGTTCGCTT	*alr8077* promoter amplification
	Palr8077R24	ACCACTACCTCCAGATCCACCC CAGCCTAATAAATCCAAATTCAT	
$p_{all3526}$CFP	Pall3526F513m	CGGCGGGGTTTTTTTTTGGAGT AATTGGGTCGTAGGTGA	*all3526* promoter amplification
	Pall3526R21	ACCACTACCTCCAGATCCACCT TCTAACTTTTCGTAGGGCAT	
p_{trn}CFP	PtrnF218m	CGGCGGGGTTTTTTTTTGGAG AGTGAATCTGAGTAATTGACTT C	*trn* promoter amplification
	PtrnR2m	CCAGCTTGCCATGCTACACCTC CTCGTTGGGTGGCAATTATTTA G	

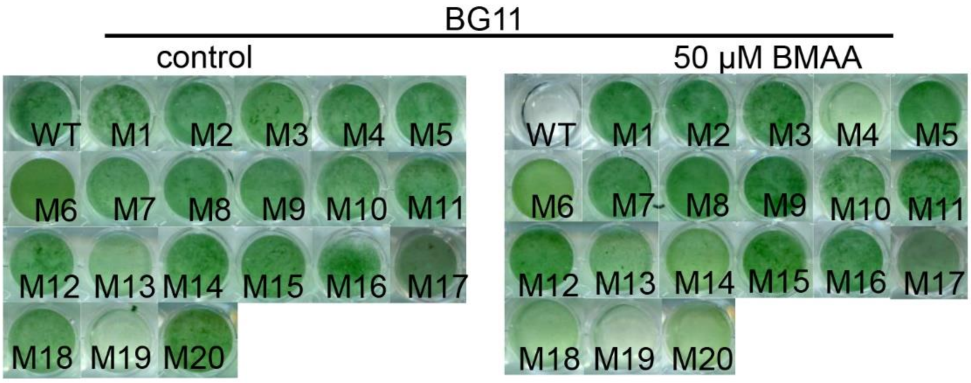

Supplementary Figure 1. BMAA resistance test in mutants M1 to M20. All strains were grown in the 24-well plates in the absence (control) or presence of 50 μM BMAA. Note that M1-M17 has been characterized in our previous papers[3,4].

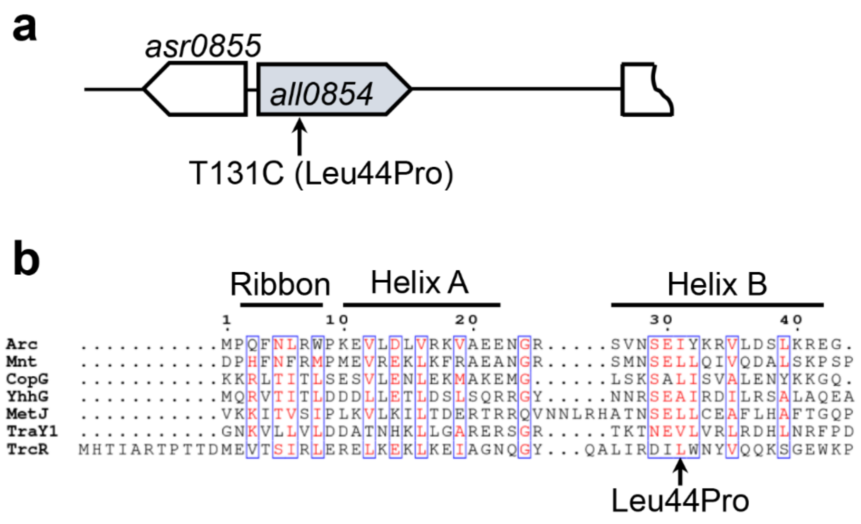

Supplementary Figure 2. The mutation site in *all0854* of M20. (a) Sketch map showing the mutation site in *all0854* of M20 identified with whole genome resequencing. (b) Sequence alignment of the DNA binding domain of some RHH family proteins. The RHH family proteins were chosen according to the work from Peter T. Chivers and Robert T. Sauer[5].

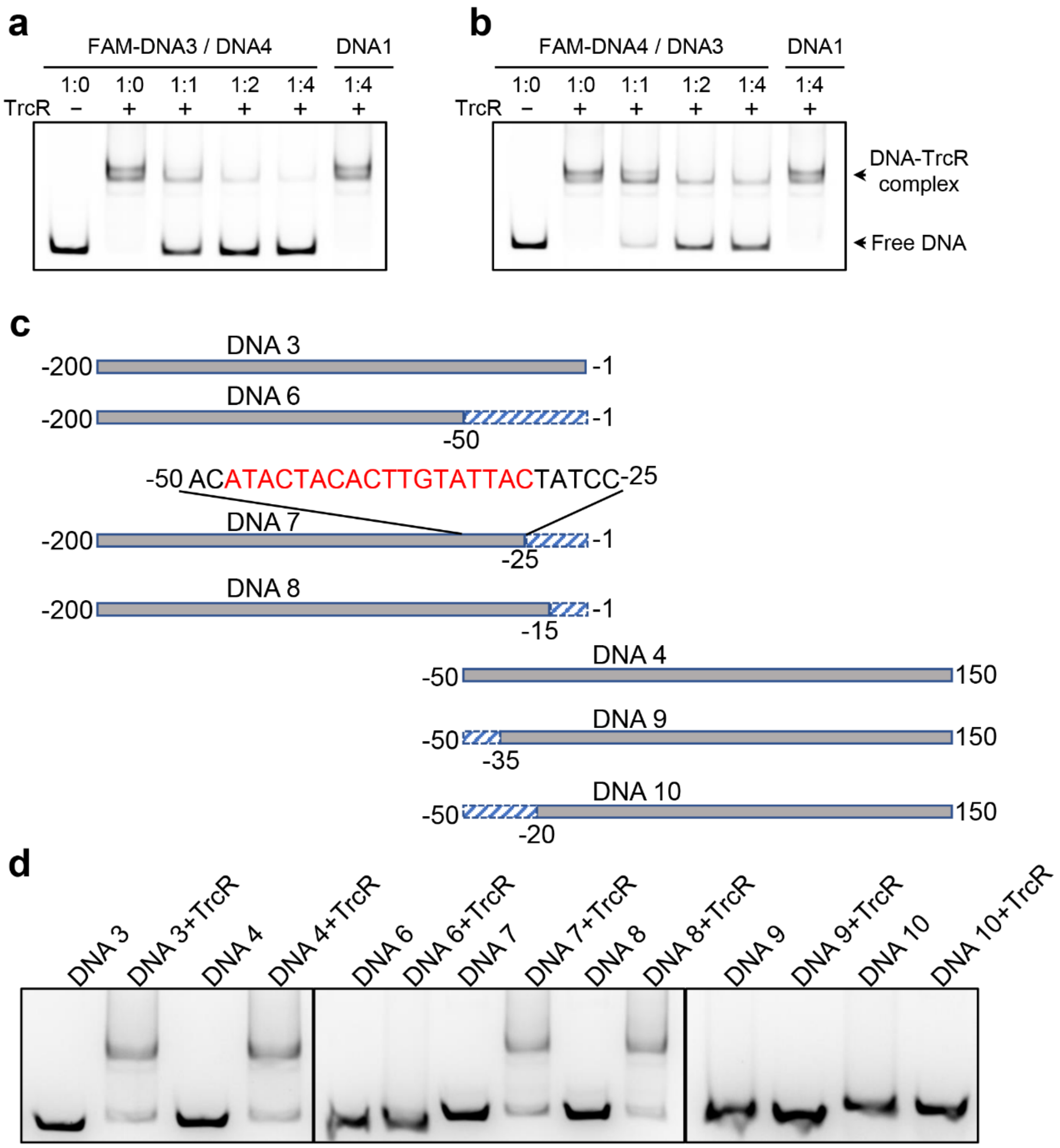

Supplementary Figure 3. EMSA test of TrcR binding to DNA3 and DNA4 specifically. (a) unlabeled DNA4 competes the binding of TrcR with FAM-labeled DNA3. (b) unlabeled DNA3 competes the binding of TrcR with FAM-labeled DNA4. (c) Illustration showing the truncated DNA fragments. The numbers on both ends of the DNA fragments indicate the relative position to the start codon. Regions with blue shading indicate the truncated portion. Letters in red indicate the conserved motif identified in Figure 2. (d) EMSA showing the binding of TrcR to truncated DNA3 and DNA4.

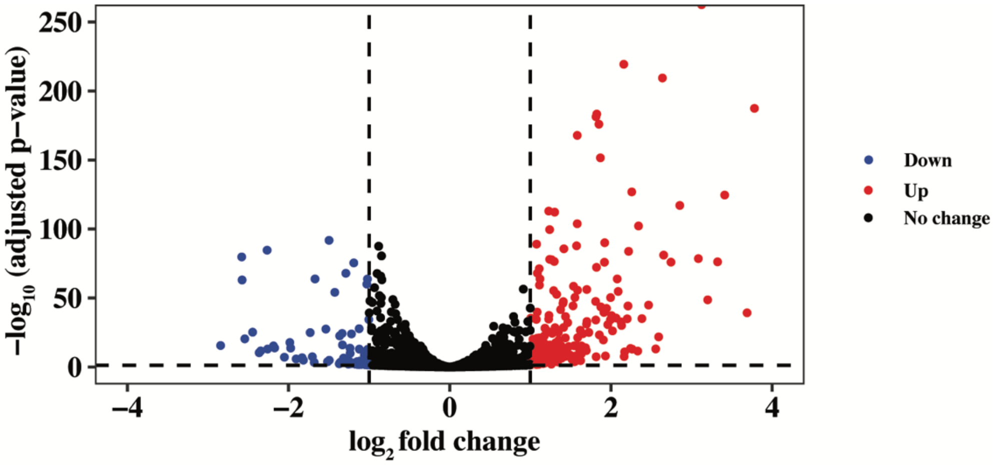

Supplementary Figure 4. Volcano plot of differentially expressed genes. Red and blue colors represent upregulated (203) and downregulated (66) genes, respectively, with a fold change > 2 and an adjusted P-value < 0.05 (indicated by dotted lines). The image was generated with ggplot2 in R.

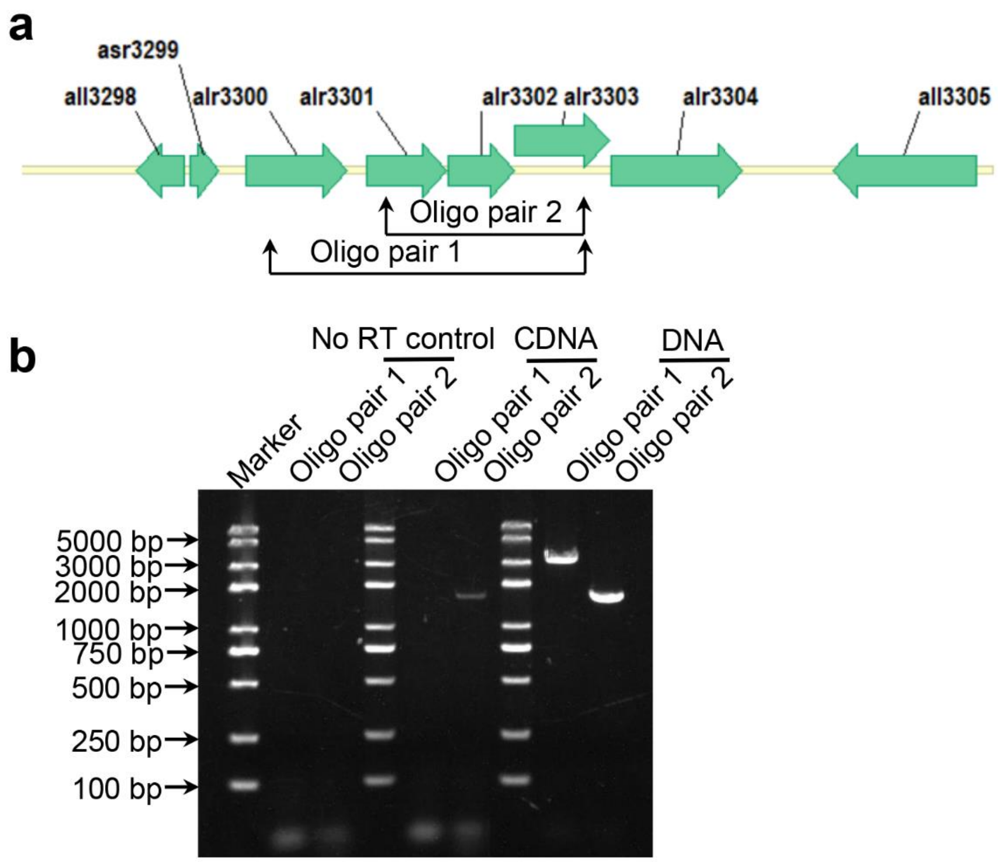

Supplementary Figure 5. *alr3301*, *alr3302* and *alr3303* constitute an operon verified by Reverse Transcription-PCR (RT-PCR). (a) Gene arrangement around *alr3301-alr3303* cluster in the genome. Oligo pairs designed for RT-PCR were indicated. (b) Agarose gel electrophoresis showing the RT-PCR results. As a control, a 3464 bp product (oligo pair 1) and a 1712 bp product (oligo pair 2) were obtained with 28 cycles of genomic DNA amplification. When using the reverse transcribed cDNA as template, a 1712 bp product was amplified by oligo pair 2, and no product obtained by oligo pair 1. Total RNA without reverse transcription (No RT control) was used as a negative control.

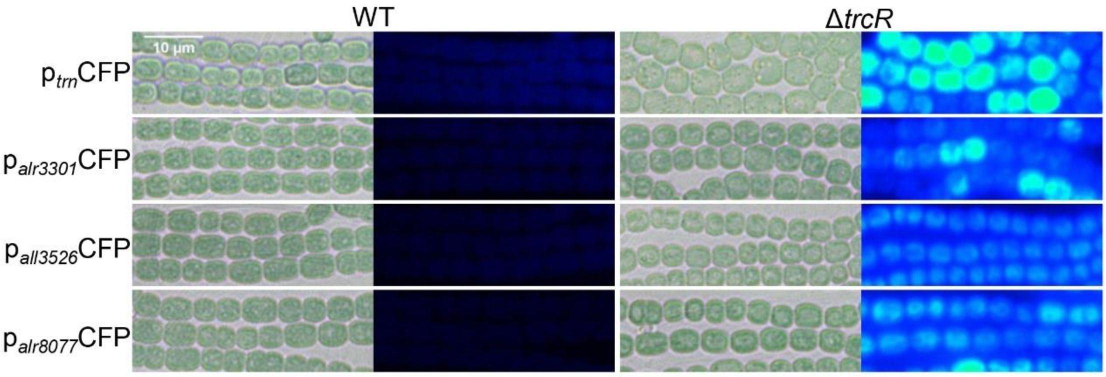

Supplementary Figure 6. Microscopic images indicating the transcription of *cfp* under specific promoters (p_{trn}CFP, $p_{alr3301}$CFP, $p_{all3526}$CFP and $p_{alr8077}$CFP) in the WT or Δ*trcR* background.

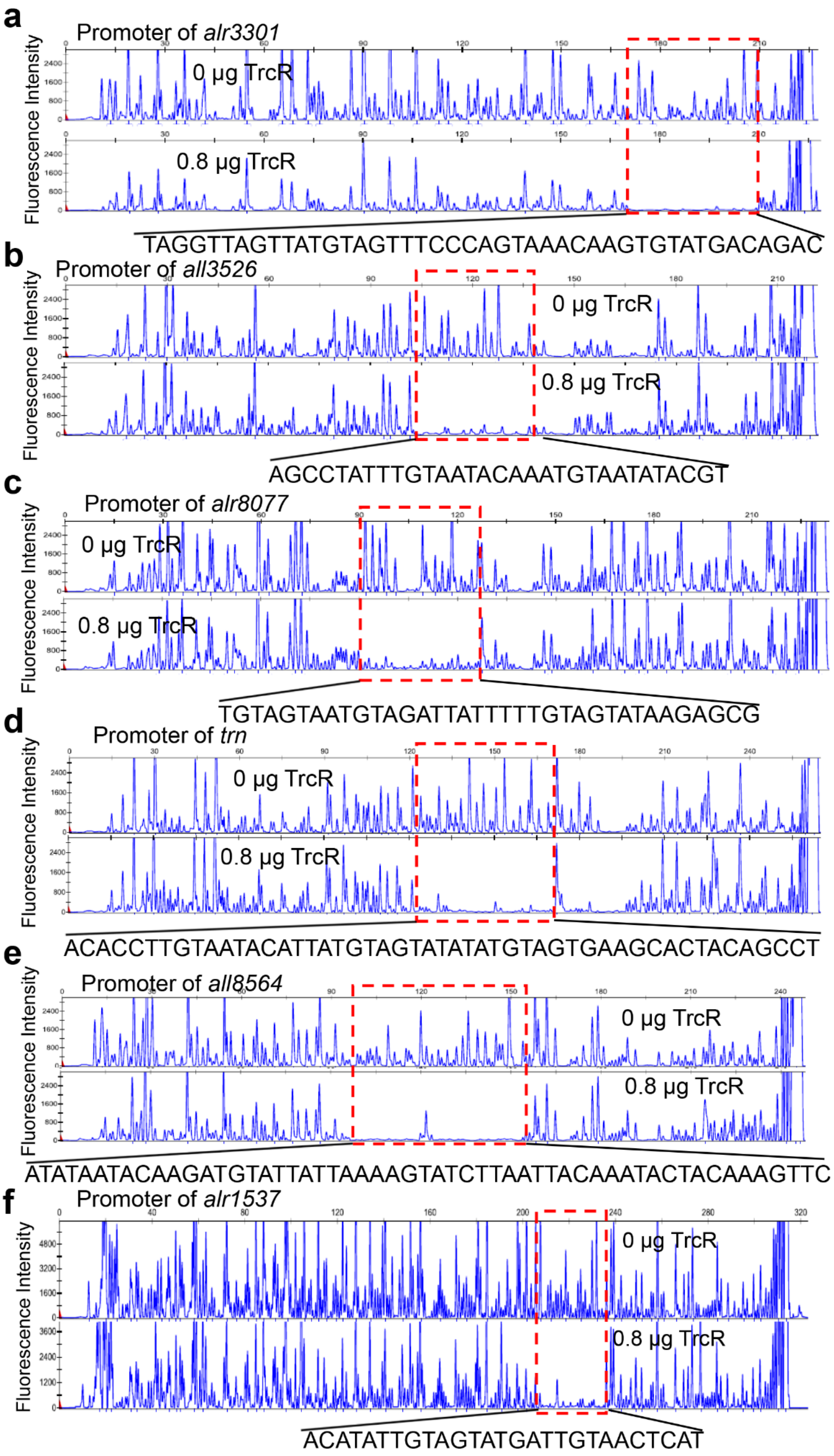

Supplementary Figure 7. DNase I footprinting assay showing the TrcR binding sequences in promoter regions

of (a) *alr3301*, (b) *all3526*, (c) *alr8077*, (d) *trn*, (e) *all8564* and (f) *alr1537*. In each figure, the upper panel shows the result without TrcR as a control and the lower panel is the result with 0.8 μg of TrcR in the reaction system. The protected region by TrcR is indicated by red frames, with corresponding sequences showing below.

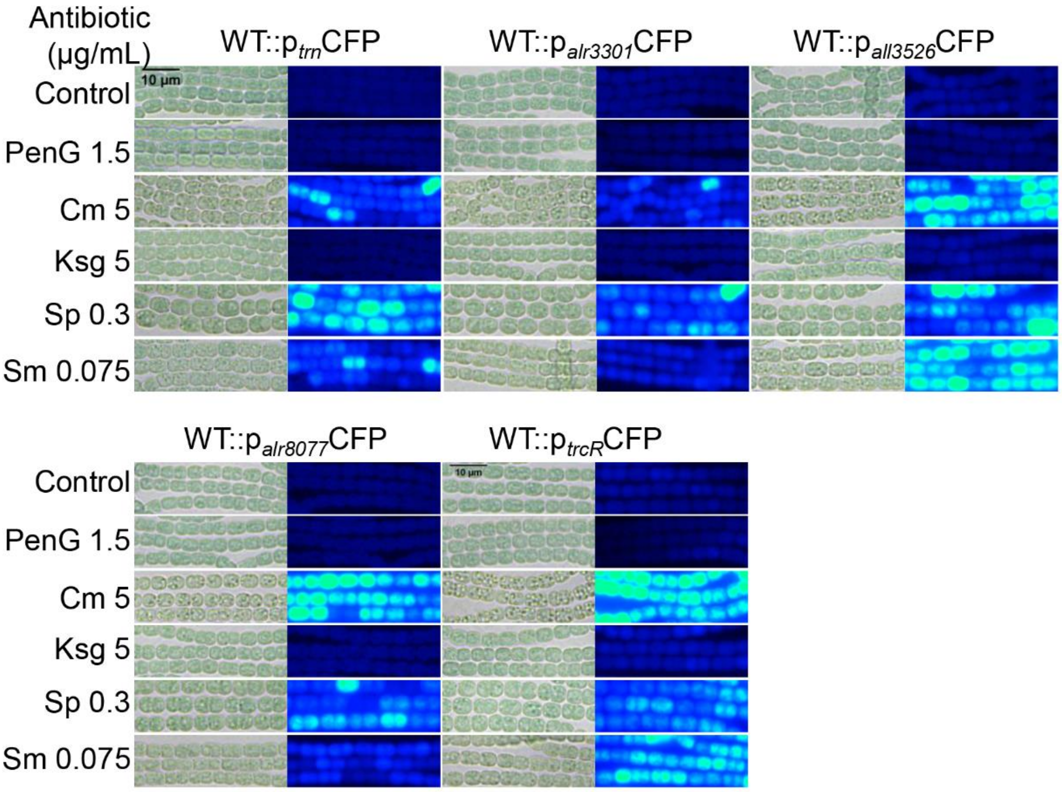

Supplementary Figure 8. Regulation of *trn*, *alr3301*, *all3526* transcription by TrcR under translational stress induced by antibiotics. The level of CFP transcription / fluorescence level indicates the level of corresponding gene expression in WT::p$_{trn}$CFP, WT::p$_{alr3301}$CFP, WT::p$_{all3526}$CFP, WT::p$_{alr8077}$CFP and WT::p$_{trcR}$CFP. Cells were incubated in BG11 containing indicated antibiotics (PenG 1.5 μg/mL, Cm 5 μg/mL, Ksg 5 μg/mL, Sp 0.3 μg/mL, Sm 0.075 μg/mL) for 48 h before imaging.

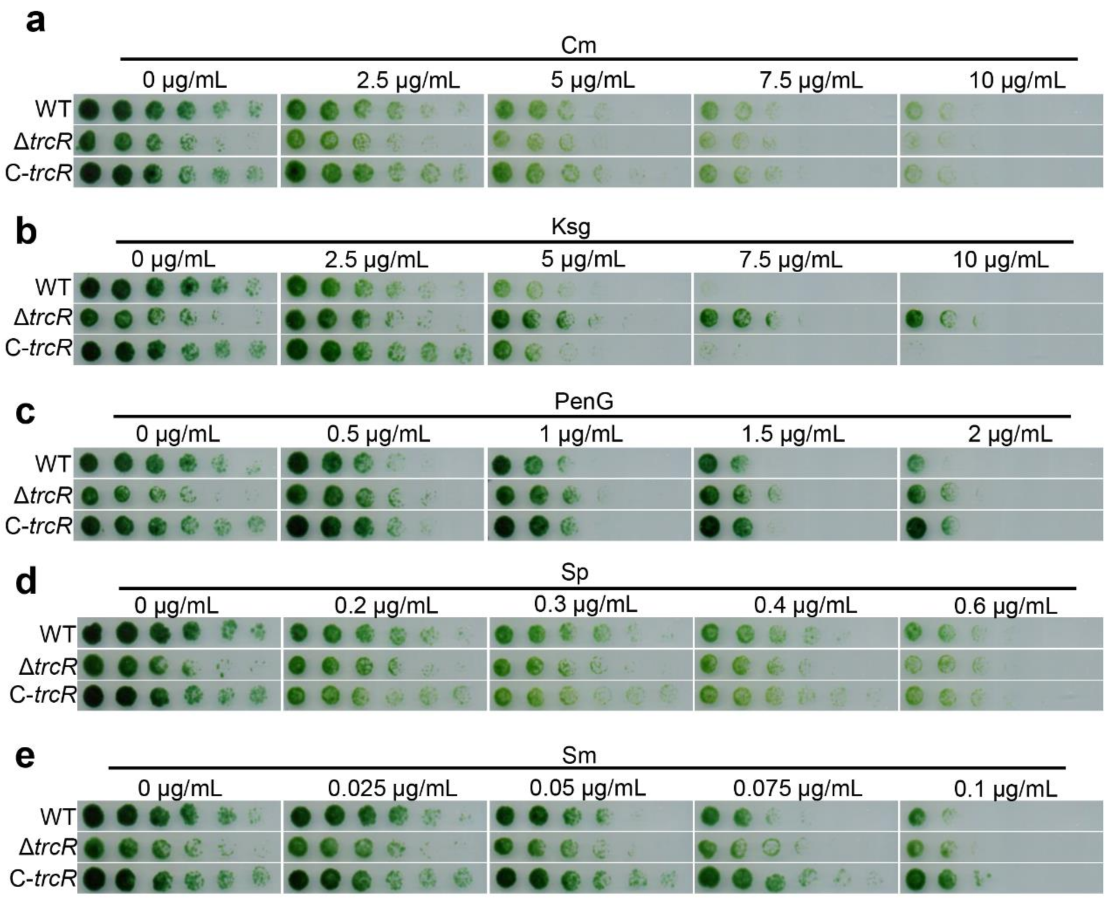

Supplementary Figure 9. Antibiotic sensitivity test. Spot assay on agar plates showing the sensitivity of WT, Δ*trcR* and C-*trcR* to different concentrations of Cm (a), Ksg (b), PenG (c), Sp (d) and Sm (e).

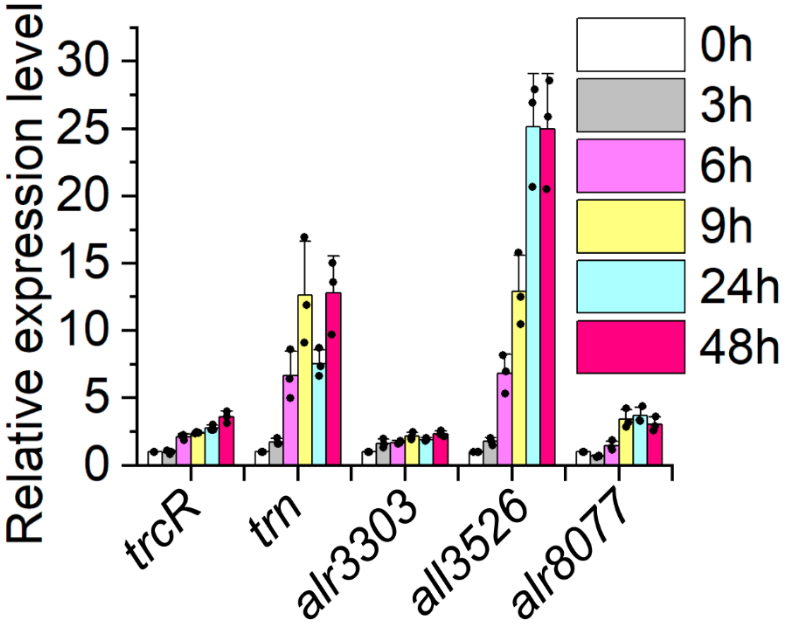

Supplementary Figure 10. Monitoring of the target gene transcription levels in the WT cells under the treatment of 5 µg/mL Cm. qRT-PCR were performed to obtain the relative transcription levels of *trcR*, *trn*, *alr3303*, *all3526* and *alr8077* at indicated time points. The transcription level of *trn* operon was indicated by an interval fragment *int* between tRNAs (see Figure 5c). n=3 biologically independent samples. The experiment was repeated three times for each sample. Data shown are the mean values ± S.D.

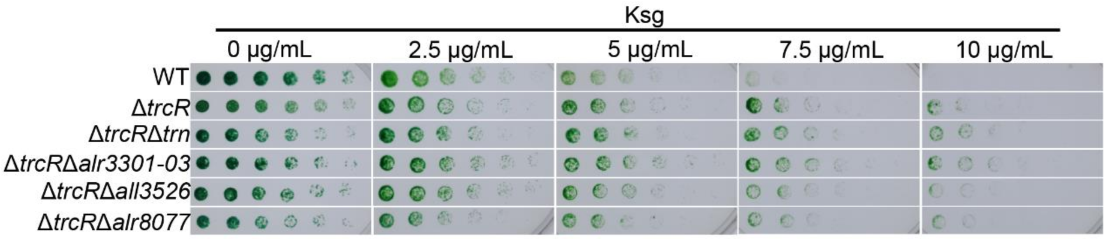

Supplementary Figure 11. Spot assay for testing Ksg sensitivity of WT and mutant strains. The sensitivity of WT, Δ*trcR*, Δ*trcR*Δ*trn*, Δ*trcR*Δ*alr3301-03*, Δ*trcR*Δ*all3526* and Δ*trcR*Δ*alr8077* to Ksg was tested at indicated concentrations.

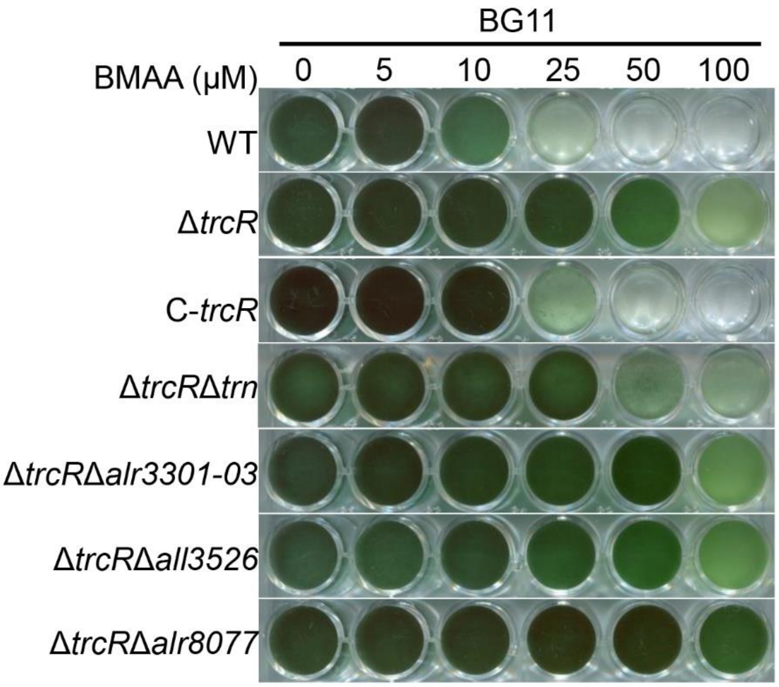

Supplementary Figure 12. BMAA sensitivity test. WT, Δ*trcR*, Δ*trcR*Δ*trn*, Δ*trcR*Δ*alr3301-03*, Δ*trcR*Δ*all3526* and Δ*trcR*Δ*alr8077* were grown in BG11 containing indicated concentrations of BMAA in 24-well plates.

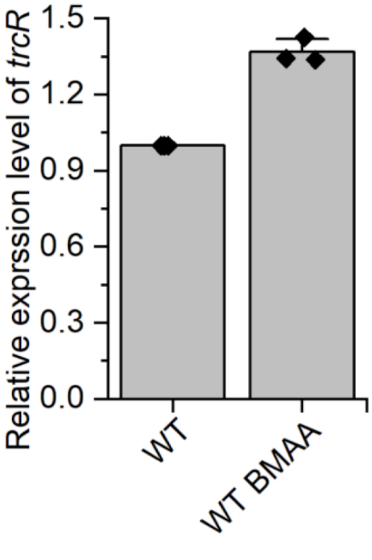

Supplementary Figure 13. Transcription levels of *trcR* quantified by qRT-PCR in WT with or without BMAA treatment. n=3 biologically independent samples. The experiment was repeated three times for each sample. Data shown are the mean values ± S.D.

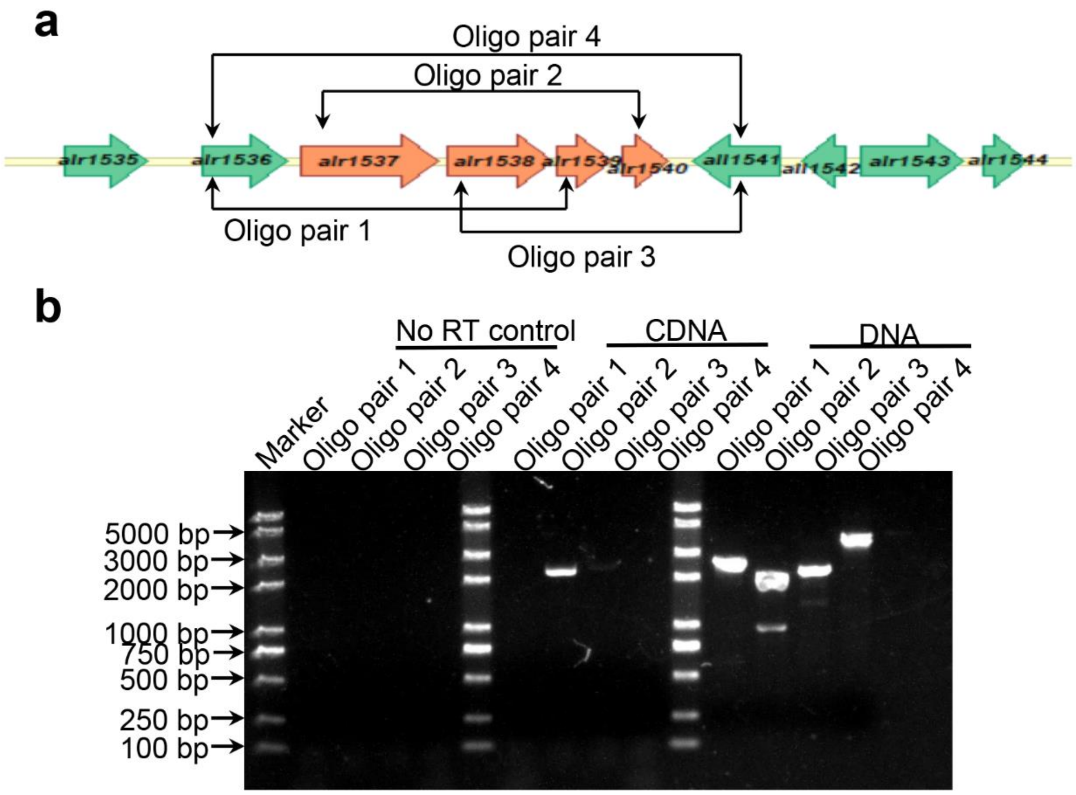

Supplementary Figure 14. Co-transcription of *alr1537*, *alr1538*, *alr1539* and *alr1540* verified by Reverse Transcription-PCR. (a) Arrangement of genes around *alr1538* on chromosome and the relative positions of the PCR products amplified by oligo pair 1, 2, 3 and 4. (b) Agarose gel electrophoresis showing the Reverse Transcription-PCR results. DNA products of 2690 bp, 2519 bp, 2651 bp and 4193 bp were obtained with 28 cycles of PCR amplification with respective oligo pairs and the genomic DNA as template. When using cDNA as template, only the 2519 bp product amplified by oligo pair 2 was obtained. RNA without reverse transcription (No RT control) was used as control.

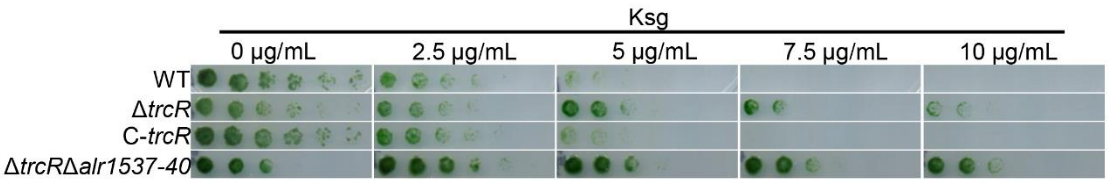

Supplementary Figure 15. Spot assay for testing Ksg sensitivity of WT, Δ*trcR*, C-*trcR* and Δ*trcR*Δ*alr1537-40*. Ksg were tested at indicated concentrations.

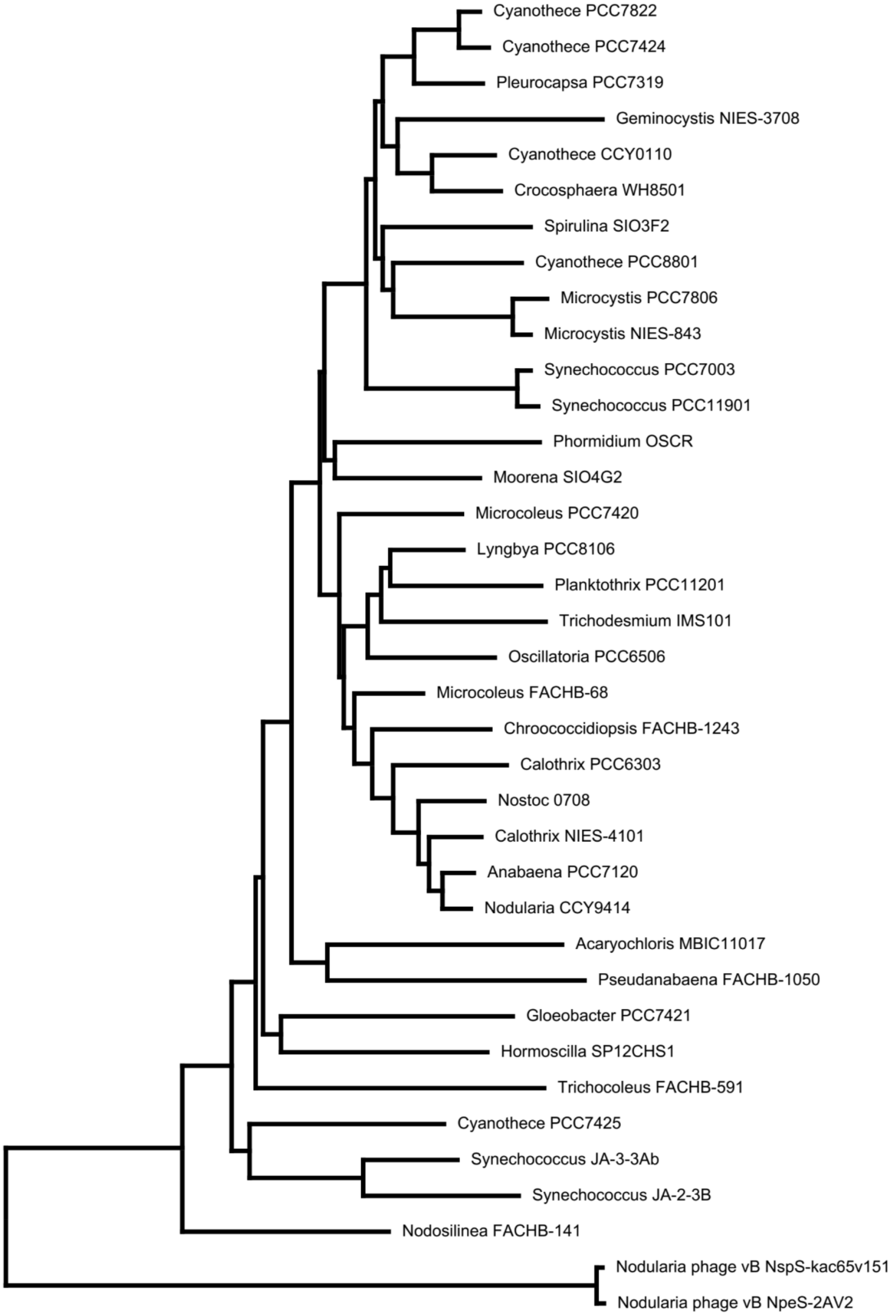

Supplementary Figure 16. Phylogenetic analysis for TrcR homologs from representative cyanobacterial strains. The tree was constructed by MEGA using the Neighbor-Joining method[6,7].

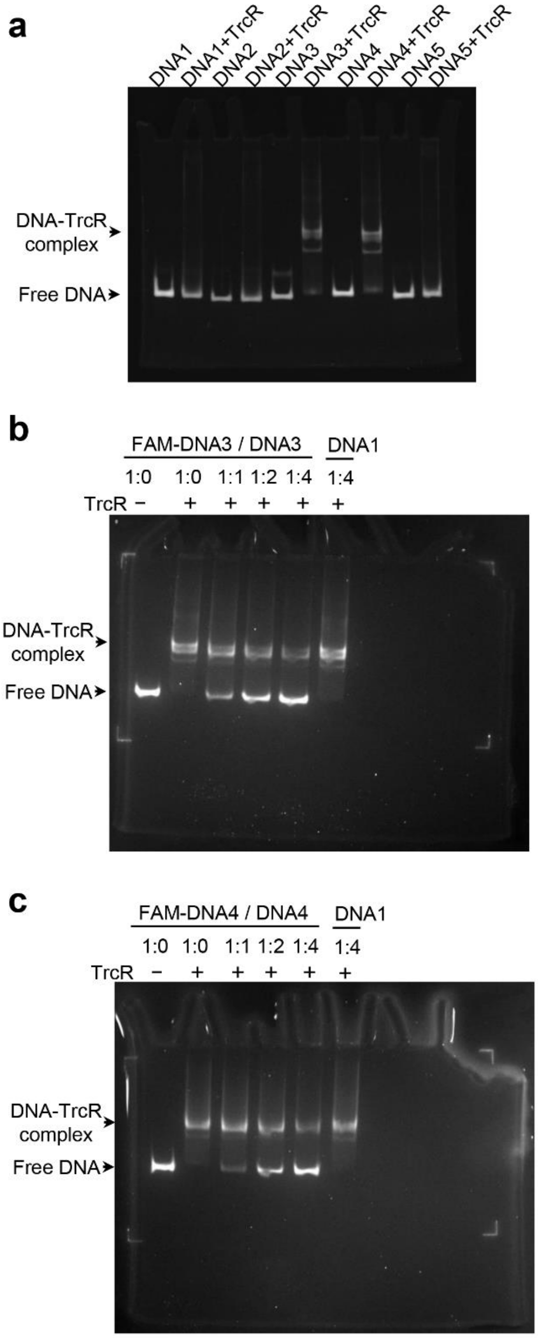

Supplementary Figure 17. The unedited gel images for Figure 2c-e. The unedited gel images for Figure 2c (a), Figure 2d (b) and Figure 2e (c).

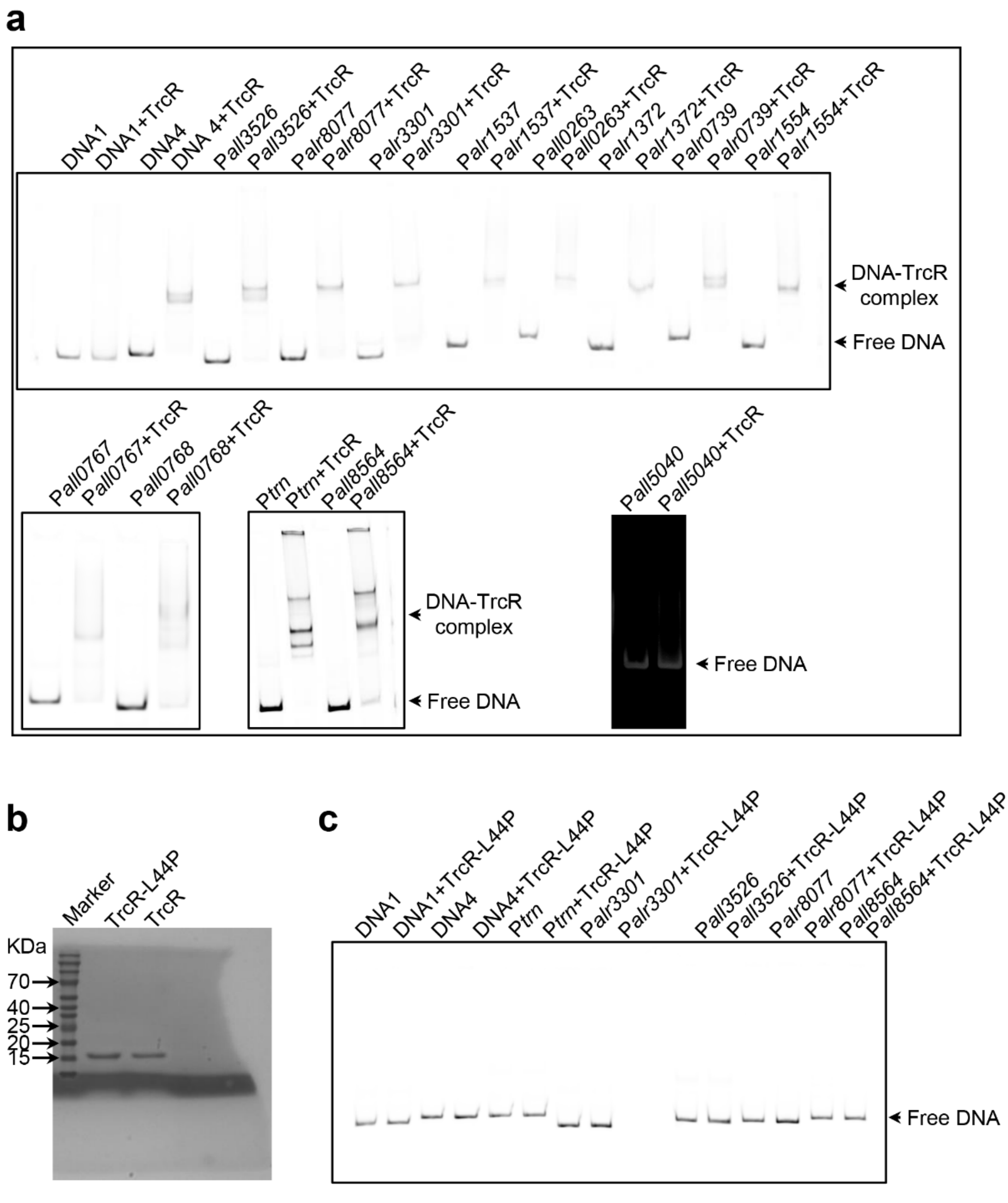

Supplementary Figure 18. The unedited gel images for Figure 4a-c. The unedited gel images for Figure 4a (a), Figure 4b (b) and Figure 4c (c).

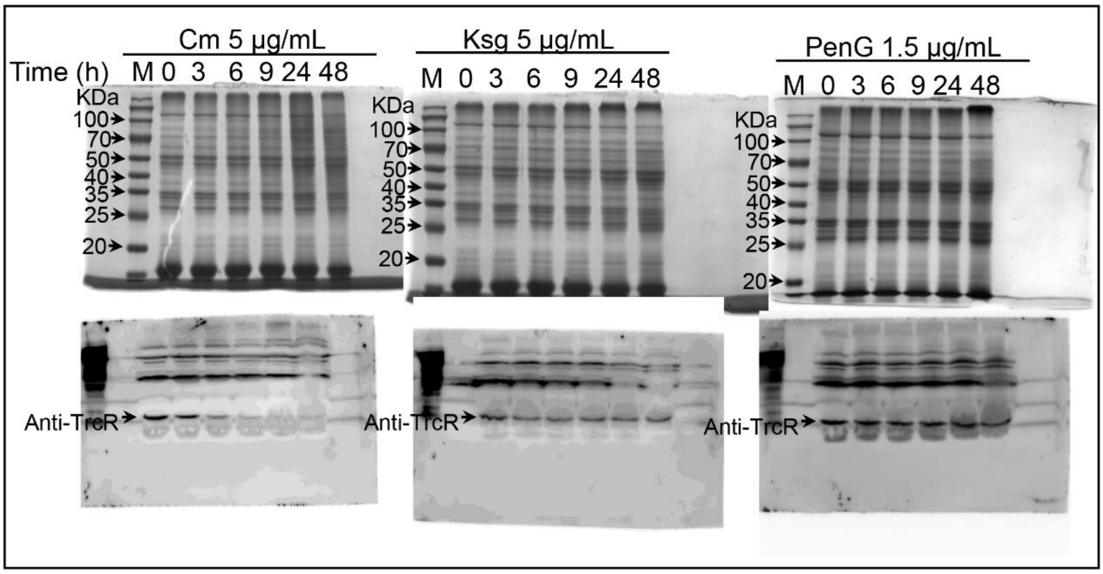

Supplementary Figure 19. The unedited blot/gel images for Figure 6b.

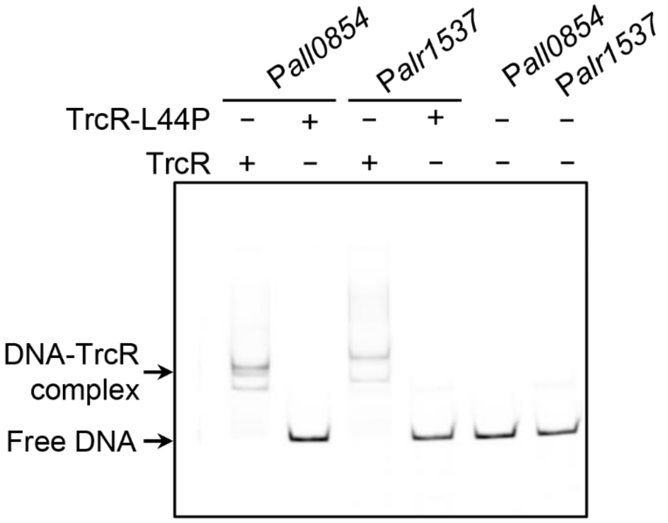

Supplementary Figure 20. The unedited gel image for Figure 8a.

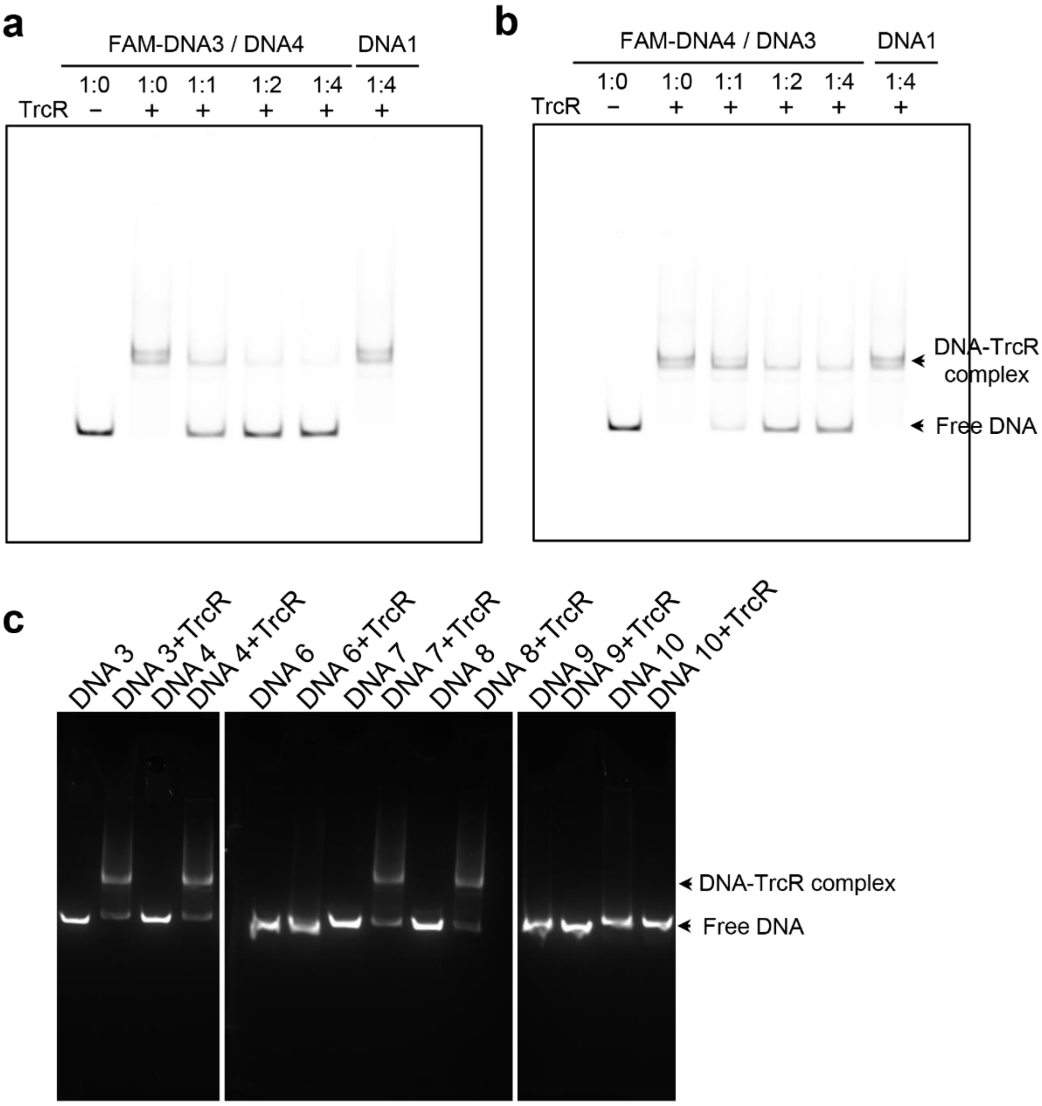

Supplementary Figure 21. The unedited gel images for Supplementary Figure 3a, 3b and 3d. The unedited gel images for Supplementary Figure 3a (a), 3b (b) and 3d (c).

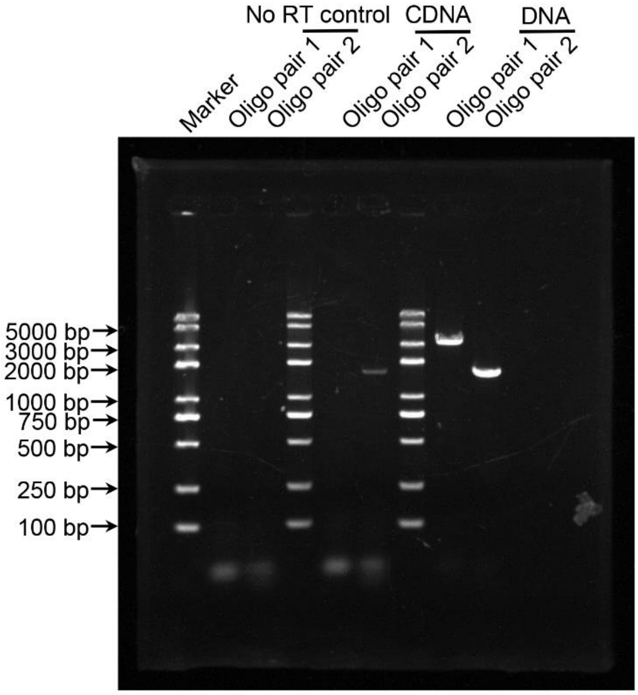

Supplementary Figure 22. The unedited gel image for Supplementary Figure 5b.

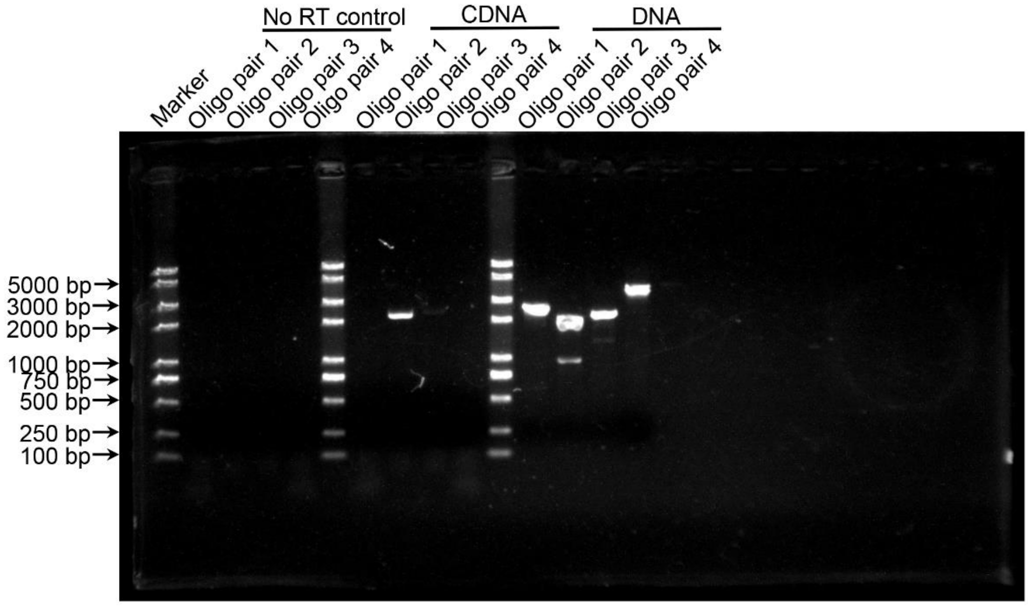

Supplementary Figure 23. The unedited gel image for Supplementary Figure 14b.

Supplementary References

1 Xing, W. Y., Xie, L. R., Zeng, X., Yang, Y. & Zhang, C. C. Functional Dissection of Genes Encoding DNA Polymerases Based on Conditional Mutants in the Heterocyst-Forming Cyanobacterium *Anabaena* PCC 7120. *Front Microbiol* **11**, 1108 (2020).

2 Santamaría-Gómez, J. *et al.* Role of a cryptic tRNA gene operon in survival under translational stress. *Nucleic Acids Res* **49**, 8757-8776 (2021).

3 Wang, Z. Q. *et al.* The Proposed Neurotoxin β-N-Methylamino-L-Alanine (BMAA) Is Taken up through Amino-Acid Transport Systems in the Cyanobacterium *Anabaena* PCC 7120. *Toxins (Basel)* **12** (2020).

4 Wang, Z. Q. & Zhang, C. C. A tRNA t(6)A modification system contributes to the sensitivity towards the toxin β-N-methylamino-L-alanine (BMAA) in the cyanobacterium *Anabaena* sp. PCC 7120. *Aquat Toxicol* **245**, 106121 (2022).

5 Chivers, P. T. & Sauer, R. T. NikR is a ribbon-helix-helix DNA-binding protein. *Protein Sci* **8**, 2494-2500 (1999).

6 Kumar, S., Stecher, G., Li, M., Knyaz, C. & Tamura, K. MEGA X: Molecular Evolutionary Genetics Analysis across Computing Platforms. *Mol Biol Evol* **35**, 1547-1549 (2018).

7 Saitou, N. & Nei, M. The neighbor-joining method: a new method for reconstructing phylogenetic trees. *Mol Biol Evol* **4**, 406-425 (1987).
