# Supplementary material for: Global translational control by the transcriptional repressor TrcR in the filamentous cyanobacterium Anabaena sp. PCC 7120
Source: Commun Biol. 2023 Jun 15;6:643. doi: 10.1038/s42003-023-05012-9 (PMC10272220; doi:10.1038/s42003-023-05012-9)
Supplement: Supplementary file 3 — Description of Additional Supplementary Files [file 42003_2023_5012_MOESM3_ESM.pdf]

Description of Additional Supplementary Files

File name: Supplementary Data 1
Description: The single nucleotide polymorphism (SNP) sites mapped in CDS (coding sequence) regions of the mutant M20 compared to WT.

File name: Supplementary Data 2
Description: The RNA-seq data analysis of genes with altered transcriptional level in ΔtrcR compared to WT.

File name: Supplementary Data 3
Description: The RNA-seq data analysis of genes with more than 2 folds changes in ΔtrcR compared to WT.

File name: Supplementary Data 4
Description: Growth curve measurement of WT, ΔtrcR, M20, C-trcR and M20-CtrcR. The source data behind Figure 1b.

File name: Supplementary Data 5
Description: Threshold cycle (Ct) values of qRT-PCR. The source data behind Figure 4d.

File name: Supplementary Data 6
Description: Threshold cycle (Ct) values of qRT-PCR. The source data behind Figure 5a, b.

File name: Supplementary Data 7
Description: The relative fluorescence intensity of WT::ptrnCFP, WT::palr3301CFP, WT::pall3526CFP, WT::palr8077CFP and WT::ptrcRCFP. The source data behind Figure 6a.

File name: Supplementary Data 8
Description: The relative integrated density (IntDen) of the TrcR band quantified from Western blot images using ImageJ. The source data behind Figure 6b.

File name: Supplementary Data 9
Description: The relative β-galactosidase activity (%) of WT and ΔtrcR acrrying different lacZ derivatives. The source data behind Figure 7.

File name: Supplementary Data 10
Description: Threshold cycle (Ct) values of qRT-PCR. The source data behind Figure 8b.

File name: Supplementary Data 11
Description: BMAA concentration in WT, ΔtrcR and ΔnatAΔbgtA at indicated time points after adding 50 µM BMAA into the medium. The source data behind Figure 8d.

File name: Supplementary Data 12
Description: Relative concentration of intracellular BMAA quantified at indicated time points after transferring the samples into BMAA-free medium. The source data behind Figure 8e.

File name: Supplementary Data 13
Description: Amount of BMAA secreted into the supernatant after transferring the samples into BMAA-free medium for 60 minutes. The source data behind Figure 8f.
